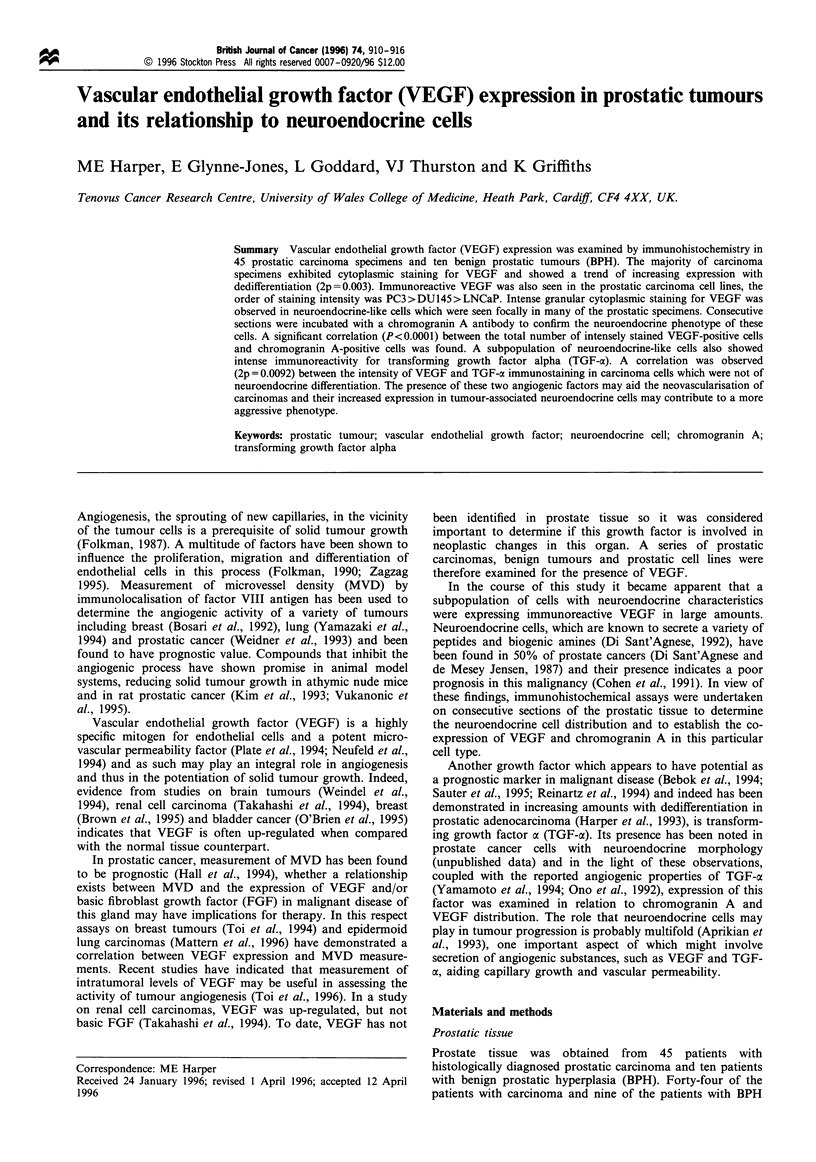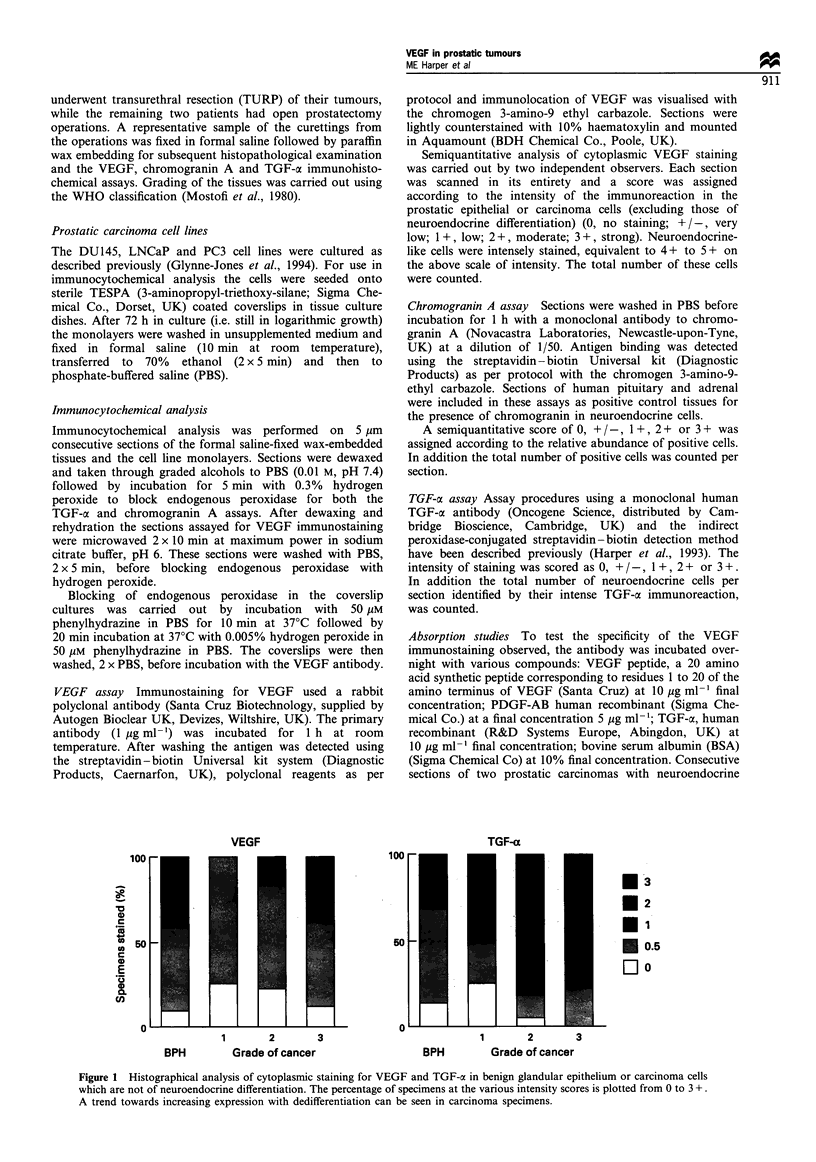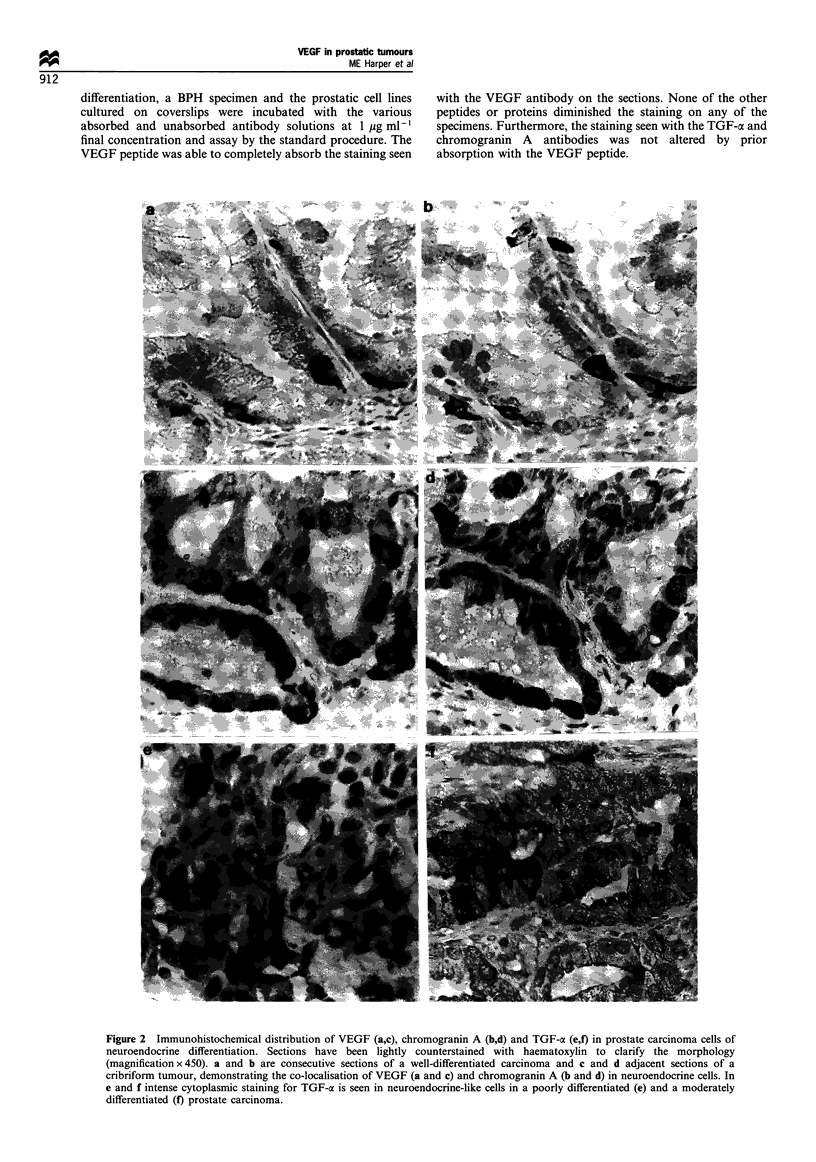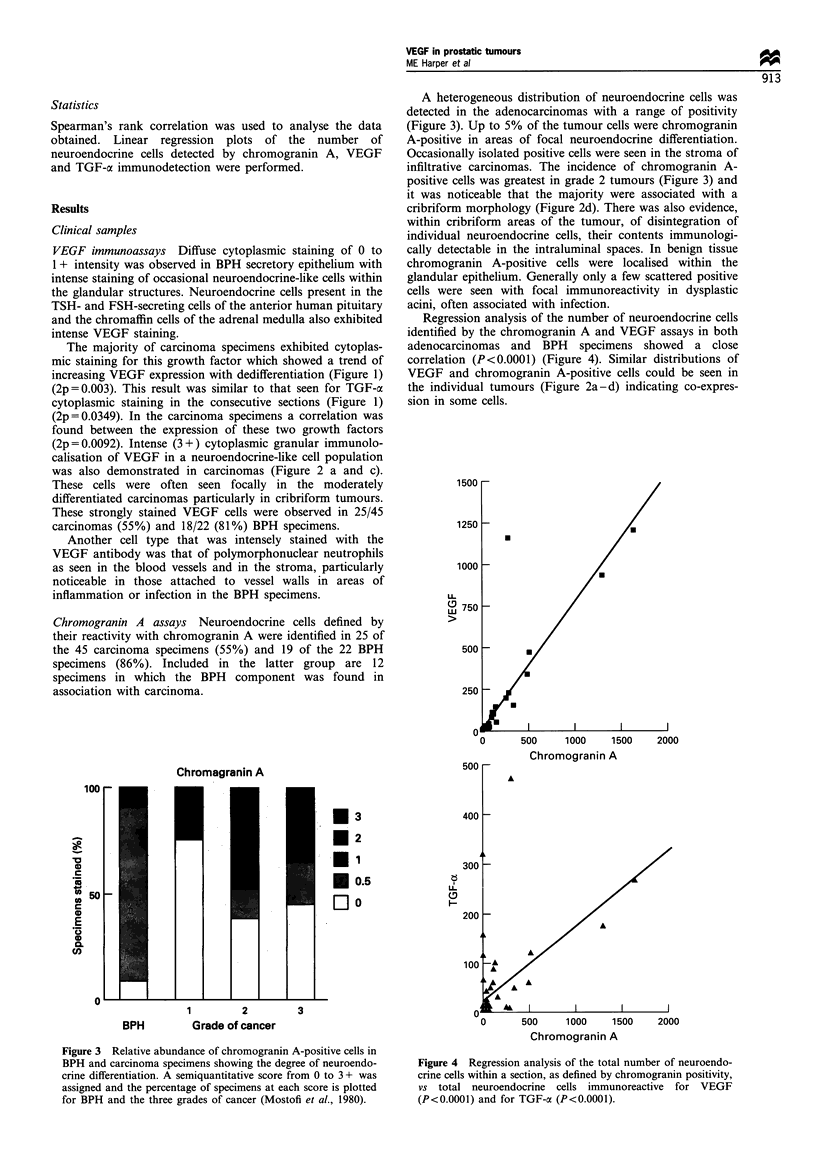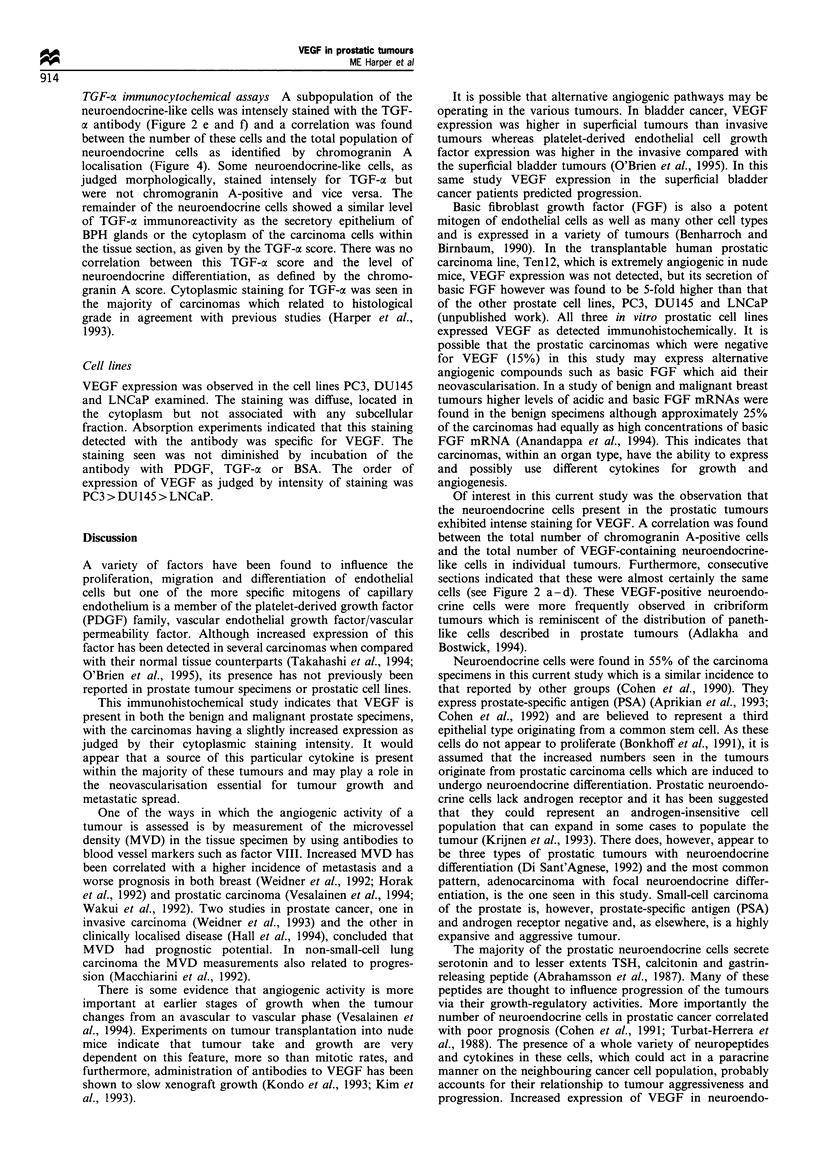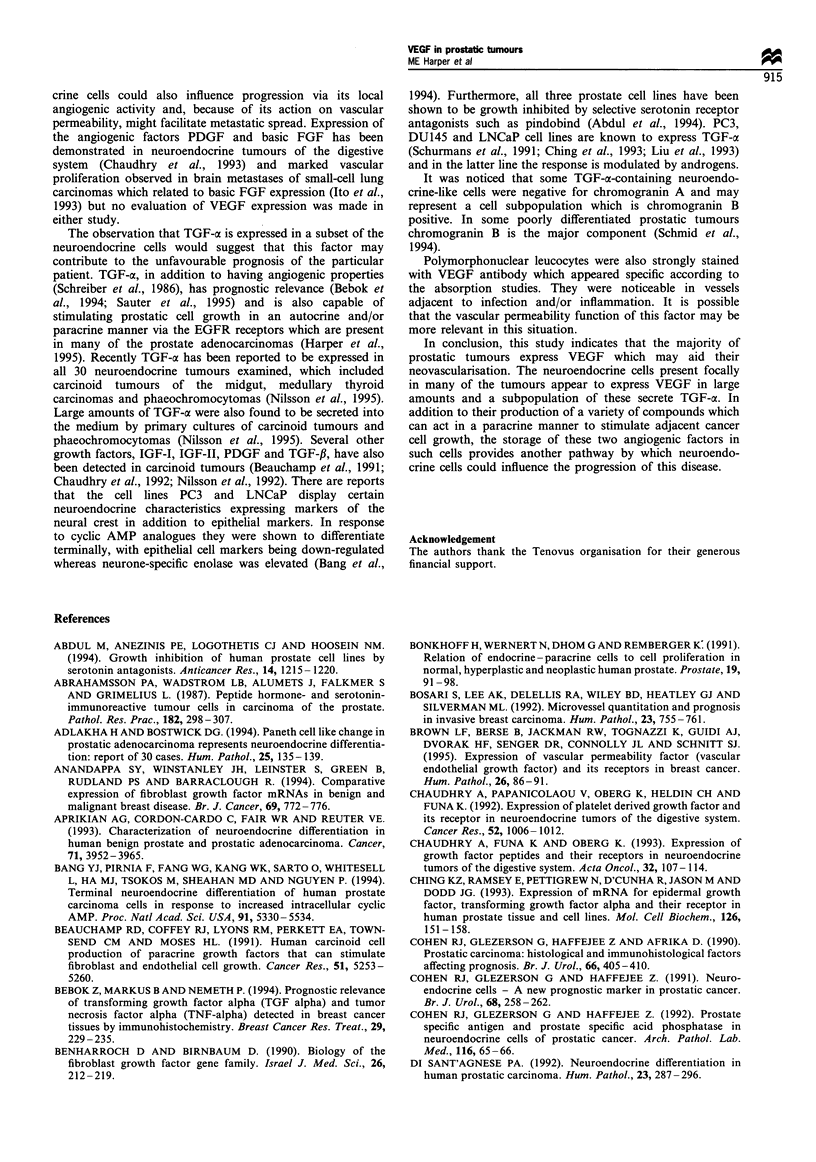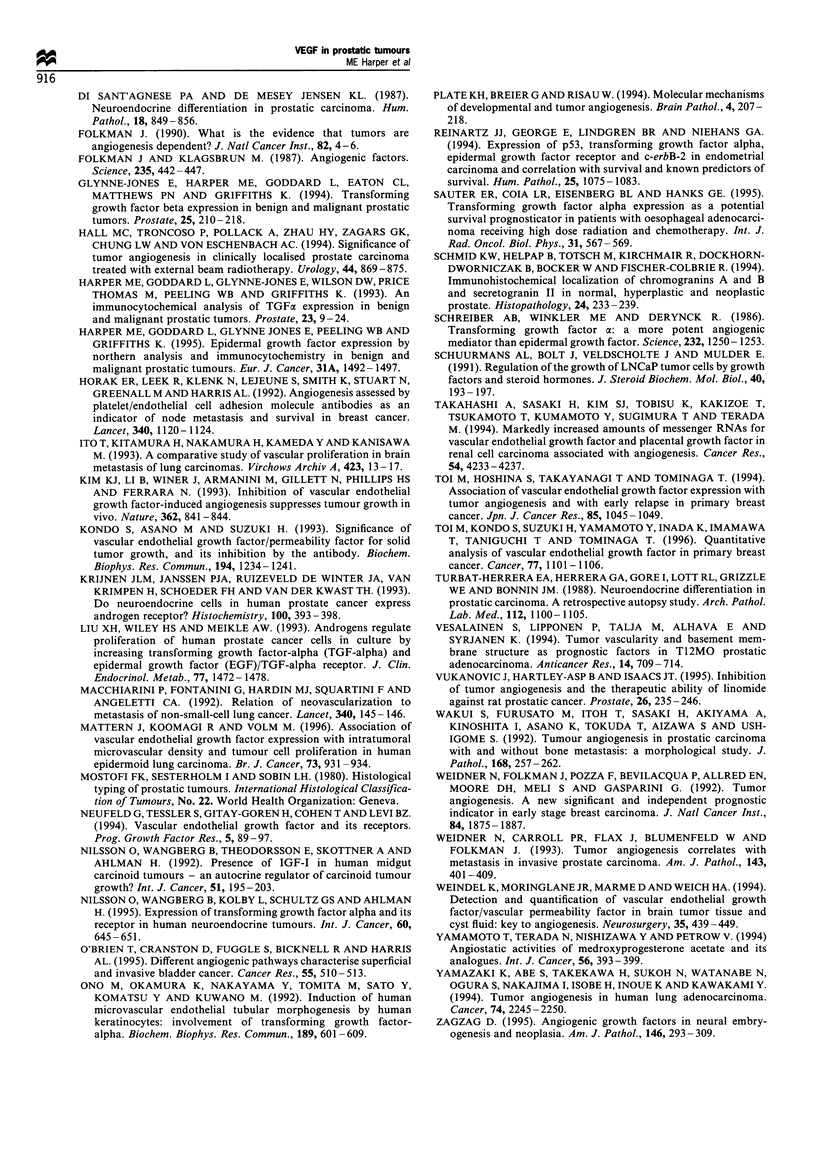# Vascular endothelial growth factor (VEGF) expression in prostatic tumours and its relationship to neuroendocrine cells.

**DOI:** 10.1038/bjc.1996.456

**Published:** 1996-09

**Authors:** M. E. Harper, E. Glynne-Jones, L. Goddard, V. J. Thurston, K. Griffiths

**Affiliations:** Tenovus Cancer Research Centre, University of Wales College of Medicine, UK.

## Abstract

**Images:**


British Journal of Cancer (1996) 74, 910-916
© 1996 Stockton Press All rights reserved 0007-0920/96 $12.00

### Vascular endothelial growth factor (VEGF) expression in prostatic tumours and its relationship to neuroendocrine cells

ME Harper, E Glynne-Jones, L Goddard, VJ Thurston and K Griffiths

*Tenovus Cancer Research Centre, University of Wales College of Medicine, Heath Park, Cardiff, CF4 4XX, UK.*

**Summary** Vascular endothelial growth factor (VEGF) expression was examined by immunohistochemistry in 45 prostatic carcinoma specimens and ten benign prostatic tumours (BPH). The majority of carcinoma specimens exhibited cytoplasmic staining for VEGF and showed a trend of increasing expression with dedifferentiation (2p = 0.003). Immunoreactive VEGF was also seen in the prostatic carcinoma cell lines, the order of staining intensity was PC3 > DU145 > LNCaP. Intense granular cytoplasmic staining for VEGF was observed in neuroendocrine-like cells which were seen focally in many of the prostatic specimens. Consecutive sections were incubated with a chromogranin A antibody to confirm the neuroendocrine phenotype of these cells. A significant correlation ($P < 0.0001$) between the total number of intensely stained VEGF-positive cells and chromogranin A-positive cells was found. A subpopulation of neuroendocrine-like cells also showed intense immunoreactivity for transforming growth factor alpha (TGF-α). A correlation was observed (2p = 0.0092) between the intensity of VEGF and TGF-α immunostaining in carcinoma cells which were not of neuroendocrine differentiation. The presence of these two angiogenic factors may aid the neovascularisation of carcinomas and their increased expression in tumour-associated neuroendocrine cells may contribute to a more aggressive phenotype.

**Keywords:** prostatic tumour; vascular endothelial growth factor; neuroendocrine cell; chromogranin A; transforming growth factor alpha

Angiogenesis, the sprouting of new capillaries, in the vicinity of the tumour cells is a prerequisite of solid tumour growth (Folkman, 1987). A multitude of factors have been shown to influence the proliferation, migration and differentiation of endothelial cells in this process (Folkman, 1990; Zagzag 1995). Measurement of microvessel density (MVD) by immunolocalisation of factor VIII antigen has been used to determine the angiogenic activity of a variety of tumours including breast (Bosari *et al.*, 1992), lung (Yamazaki *et al.*, 1994) and prostatic cancer (Weidner *et al.*, 1993) and been found to have prognostic value. Compounds that inhibit the angiogenic process have shown promise in animal model systems, reducing solid tumour growth in athymic nude mice and in rat prostatic cancer (Kim *et al.*, 1993; Vukanonic *et al.*, 1995).

Vascular endothelial growth factor (VEGF) is a highly specific mitogen for endothelial cells and a potent microvascular permeability factor (Plate *et al.*, 1994; Neufeld *et al.*, 1994) and as such may play an integral role in angiogenesis and thus in the potentiation of solid tumour growth. Indeed, evidence from studies on brain tumours (Weindel *et al.*, 1994), renal cell carcinoma (Takahashi *et al.*, 1994), breast (Brown *et al.*, 1995) and bladder cancer (O'Brien *et al.*, 1995) indicates that VEGF is often up-regulated when compared with the normal tissue counterpart.

In prostatic cancer, measurement of MVD has been found to be prognostic (Hall *et al.*, 1994), whether a relationship exists between MVD and the expression of VEGF and/or basic fibroblast growth factor (FGF) in malignant disease of this gland may have implications for therapy. In this respect assays on breast tumours (Toi *et al.*, 1994) and epidermoid lung carcinomas (Mattern *et al.*, 1996) have demonstrated a correlation between VEGF expression and MVD measurements. Recent studies have indicated that measurement of intratumoral levels of VEGF may be useful in assessing the activity of tumour angiogenesis (Toi *et al.*, 1996). In a study on renal cell carcinomas, VEGF was up-regulated, but not basic FGF (Takahashi *et al.*, 1994). To date, VEGF has not been identified in prostate tissue so it was considered important to determine if this growth factor is involved in neoplastic changes in this organ. A series of prostatic carcinomas, benign tumours and prostatic cell lines were therefore examined for the presence of VEGF.

In the course of this study it became apparent that a subpopulation of cells with neuroendocrine characteristics were expressing immunoreactive VEGF in large amounts. Neuroendocrine cells, which are known to secrete a variety of peptides and biogenic amines (Di Sant'Agnese, 1992), have been found in 50% of prostate cancers (Di Sant'Agnese and de Mesey Jensen, 1987) and their presence indicates a poor prognosis in this malignancy (Cohen *et al.*, 1991). In view of these findings, immunohistochemical assays were undertaken on consecutive sections of the prostatic tissue to determine the neuroendocrine cell distribution and to establish the co-expression of VEGF and chromogranin A in this particular cell type.

Another growth factor which appears to have potential as a prognostic marker in malignant disease (Bebok *et al.*, 1994; Sauter *et al.*, 1995; Reinartz *et al.*, 1994) and indeed has been demonstrated in increasing amounts with dedifferentiation in prostatic adenocarcinoma (Harper *et al.*, 1993), is transforming growth factor α (TGF-α). Its presence has been noted in prostate cancer cells with neuroendocrine morphology (unpublished data) and in the light of these observations, coupled with the reported angiogenic properties of TGF-α (Yamamoto *et al.*, 1994; Ono *et al.*, 1992), expression of this factor was examined in relation to chromogranin A and VEGF distribution. The role that neuroendocrine cells may play in tumour progression is probably multifold (Aprikian *et al.*, 1993), one important aspect of which might involve secretion of angiogenic substances, such as VEGF and TGF-α, aiding capillary growth and vascular permeability.

### Materials and methods

### *Prostatic tissue*

Prostate tissue was obtained from 45 patients with histologically diagnosed prostatic carcinoma and ten patients with benign prostatic hyperplasia (BPH). Forty-four of the patients with carcinoma and nine of the patients with BPH

Correspondence: ME Harper
Received 24 January 1996; revised 1 April 1996; accepted 12 April 1996

underwent transurethral resection (TURP) of their tumours, while the remaining two patients had open prostatectomy operations. A representative sample of the curettings from the operations was fixed in formal saline followed by paraffin wax embedding for subsequent histopathological examination and the VEGF, chromogranin A and TGF-α immunohistochemical assays. Grading of the tissues was carried out using the WHO classification (Mostofi *et al*., 1980).

### *Prostatic carcinoma cell lines*

The DU145, LNCaP and PC3 cell lines were cultured as described previously (Glynne-Jones *et al*., 1994). For use in immunocytochemical analysis the cells were seeded onto sterile TESPA (3-aminopropyl-triethoxy-silane; Sigma Chemical Co., Dorset, UK) coated coverslips in tissue culture dishes. After 72 h in culture (i.e. still in logarithmic growth) the monolayers were washed in unsupplemented medium and fixed in formal saline (10 min at room temperature), transferred to 70% ethanol (2 × 5 min) and then to phosphate-buffered saline (PBS).

### *Immunocytochemical analysis*

Immunocytochemical analysis was performed on 5 μm consecutive sections of the formal saline-fixed wax-embedded tissues and the cell line monolayers. Sections were dewaxed and taken through graded alcohols to PBS (0.01 M, pH 7.4) followed by incubation for 5 min with 0.3% hydrogen peroxide to block endogenous peroxidase for both the TGF-α and chromogranin A assays. After dewaxing and rehydration the sections assayed for VEGF immunostaining were microwaved 2 × 10 min at maximum power in sodium citrate buffer, pH 6. These sections were washed with PBS, 2 × 5 min, before blocking endogenous peroxidase with hydrogen peroxide.

Blocking of endogenous peroxidase in the coverslip cultures was carried out by incubation with 50 μM phenylhydrazine in PBS for 10 min at 37°C followed by 20 min incubation at 37°C with 0.005% hydrogen peroxide in 50 μM phenylhydrazine in PBS. The coverslips were then washed, 2 × PBS, before incubation with the VEGF antibody.

*VEGF assay* Immunostaining for VEGF used a rabbit polyclonal antibody (Santa Cruz Biotechnology, supplied by Autogen Bioclear UK, Devizes, Wiltshire, UK). The primary antibody (1 μg $ml^{-1}$) was incubated for 1 h at room temperature. After washing the antigen was detected using the streptavidin–biotin Universal kit system (Diagnostic Products, Caernarfon, UK), polyclonal reagents as per protocol and immunolocation of VEGF was visualised with the chromogen 3-amino-9 ethyl carbazole. Sections were lightly counterstained with 10% haematoxylin and mounted in Aquamount (BDH Chemical Co., Poole, UK).

Semiquantitative analysis of cytoplasmic VEGF staining was carried out by two independent observers. Each section was scanned in its entirety and a score was assigned according to the intensity of the immunoreaction in the prostatic epithelial or carcinoma cells (excluding those of neuroendocrine differentiation) (0, no staining; +/−, very low; 1+, low; 2+, moderate; 3+, strong). Neuroendocrine-like cells were intensely stained, equivalent to 4+ to 5+ on the above scale of intensity. The total number of these cells were counted.

*Chromogranin A assay* Sections were washed in PBS before incubation for 1 h with a monoclonal antibody to chromogranin A (Novacastra Laboratories, Newcastle-upon-Tyne, UK) at a dilution of 1/50. Antigen binding was detected using the streptavidin–biotin Universal kit (Diagnostic Products) as per protocol with the chromogen 3-amino-9-ethyl carbazole. Sections of human pituitary and adrenal were included in these assays as positive control tissues for the presence of chromogranin in neuroendocrine cells.

A semiquantitative score of 0, +/−, 1+, 2+ or 3+ was assigned according to the relative abundance of positive cells. In addition the total number of positive cells was counted per section.

*TGF-α assay* Assay procedures using a monoclonal human TGF-α antibody (Oncogene Science, distributed by Cambridge Bioscience, Cambridge, UK) and the indirect peroxidase-conjugated streptavidin–biotin detection method have been described previously (Harper *et al*., 1993). The intensity of staining was scored as 0, +/−, 1+, 2+ or 3+. In addition the total number of neuroendocrine cells per section identified by their intense TGF-α immunoreaction, was counted.

*Absorption studies* To test the specificity of the VEGF immunostaining observed, the antibody was incubated overnight with various compounds: VEGF peptide, a 20 amino acid synthetic peptide corresponding to residues 1 to 20 of the amino terminus of VEGF (Santa Cruz) at 10 μg $ml^{-1}$ final concentration; PDGF-AB human recombinant (Sigma Chemical Co.) at a final concentration 5 μg $ml^{-1}$; TGF-α, human recombinant (R&D Systems Europe, Abingdon, UK) at 10 μg $ml^{-1}$ final concentration; bovine serum albumin (BSA) (Sigma Chemical Co) at 10% final concentration. Consecutive sections of two prostatic carcinomas with neuroendocrine

**Figure 1** Histographical analysis of cytoplasmic staining for VEGF and TGF-α in benign glandular epithelium or carcinoma cells which are not of neuroendocrine differentiation. The percentage of specimens at the various intensity scores is plotted from 0 to 3+. A trend towards increasing expression with dedifferentiation can be seen in carcinoma specimens.

differentiation, a BPH specimen and the prostatic cell lines cultured on coverslips were incubated with the various absorbed and unabsorbed antibody solutions at 1 $\mu$g ml$^{-1}$ final concentration and assay by the standard procedure. The VEGF peptide was able to completely absorb the staining seen with the VEGF antibody on the sections. None of the other peptides or proteins diminished the staining on any of the specimens. Furthermore, the staining seen with the TGF-$\alpha$ and chromogranin A antibodies was not altered by prior absorption with the VEGF peptide.

**Figure 2** Immunohistochemical distribution of VEGF (**a**,**c**), chromogranin A (**b**,**d**) and TGF-$\alpha$ (**e**,**f**) in prostate carcinoma cells of neuroendocrine differentiation. Sections have been lightly counterstained with haematoxylin to clarify the morphology (magnification $\times$ 450). **a** and **b** are consecutive sections of a well-differentiated carcinoma and **c** and **d** adjacent sections of a cribriform tumour, demonstrating the co-localisation of VEGF (**a** and **c**) and chromogranin A (**b** and **d**) in neuroendocrine cells. In **e** and **f** intense cytoplasmic staining for TGF-$\alpha$ is seen in neuroendocrine-like cells in a poorly differentiated (**e**) and a moderately differentiated (**f**) prostate carcinoma.

*Statistics*

Spearman's rank correlation was used to analyse the data obtained. Linear regression plots of the number of neuroendocrine cells detected by chromogranin A, VEGF and TGF-α immunodetection were performed.

### Results

*Clinical samples*

*VEGF immunoassays* Diffuse cytoplasmic staining of 0 to 1+ intensity was observed in BPH secretory epithelium with intense staining of occasional neuroendocrine-like cells within the glandular structures. Neuroendocrine cells present in the TSH- and FSH-secreting cells of the anterior human pituitary and the chromaffin cells of the adrenal medulla also exhibited intense VEGF staining.

The majority of carcinoma specimens exhibited cytoplasmic staining for this growth factor which showed a trend of increasing VEGF expression with dedifferentiation (Figure 1) (2p = 0.003). This result was similar to that seen for TGF-α cytoplasmic staining in the consecutive sections (Figure 1) (2p = 0.0349). In the carcinoma specimens a correlation was found between the expression of these two growth factors (2p = 0.0092). Intense (3+) cytoplasmic granular immunolocalisation of VEGF in a neuroendocrine-like cell population was also demonstrated in carcinomas (Figure 2 a and c). These cells were often seen focally in the moderately differentiated carcinomas particularly in cribriform tumours. These strongly stained VEGF cells were observed in 25/45 carcinomas (55%) and 18/22 (81%) BPH specimens.

Another cell type that was intensely stained with the VEGF antibody was that of polymorphonuclear neutrophils as seen in the blood vessels and in the stroma, particularly noticeable in those attached to vessel walls in areas of inflammation or infection in the BPH specimens.

*Chromogranin A assays* Neuroendocrine cells defined by their reactivity with chromogranin A were identified in 25 of the 45 carcinoma specimens (55%) and 19 of the 22 BPH specimens (86%). Included in the latter group are 12 specimens in which the BPH component was found in association with carcinoma.

**Figure 3** Relative abundance of chromogranin A-positive cells in BPH and carcinoma specimens showing the degree of neuroendocrine differentiation. A semiquantitative score from 0 to 3+ was assigned and the percentage of specimens at each score is plotted for BPH and the three grades of cancer (Mostofi *et al*., 1980).

A heterogeneous distribution of neuroendocrine cells was detected in the adenocarcinomas with a range of positivity (Figure 3). Up to 5% of the tumour cells were chromogranin A-positive in areas of focal neuroendocrine differentiation. Occasionally isolated positive cells were seen in the stroma of infiltrative carcinomas. The incidence of chromogranin A-positive cells was greatest in grade 2 tumours (Figure 3) and it was noticeable that the majority were associated with a cribriform morphology (Figure 2d). There was also evidence, within cribriform areas of the tumour, of disintegration of individual neuroendocrine cells, their contents immunologically detectable in the intraluminal spaces. In benign tissue chromogranin A-positive cells were localised within the glandular epithelium. Generally only a few scattered positive cells were seen with focal immunoreactivity in dysplastic acini, often associated with infection.

Regression analysis of the number of neuroendocrine cells identified by the chromogranin A and VEGF assays in both adenocarcinomas and BPH specimens showed a close correlation ($P<0.0001$) (Figure 4). Similar distributions of VEGF and chromogranin A-positive cells could be seen in the individual tumours (Figure 2a–d) indicating co-expression in some cells.

**Figure 4** Regression analysis of the total number of neuroendocrine cells within a section, as defined by chromogranin positivity, *vs* total neuroendocrine cells immunoreactive for VEGF ($P<0.0001$) and for TGF-α ($P<0.0001$).

*TGF-α immunocytochemical assays* A subpopulation of the neuroendocrine-like cells was intensely stained with the TGF-α antibody (Figure 2 e and f) and a correlation was found between the number of these cells and the total population of neuroendocrine cells as identified by chromogranin A localisation (Figure 4). Some neuroendocrine-like cells, as judged morphologically, stained intensely for TGF-α but were not chromogranin A-positive and vice versa. The remainder of the neuroendocrine cells showed a similar level of TGF-α immunoreactivity as the secretory epithelium of BPH glands or the cytoplasm of the carcinoma cells within the tissue section, as given by the TGF-α score. There was no correlation between this TGF-α score and the level of neuroendocrine differentiation, as defined by the chromogranin A score. Cytoplasmic staining for TGF-α was seen in the majority of carcinomas which related to histological grade in agreement with previous studies (Harper *et al.*, 1993).

*Cell lines*

VEGF expression was observed in the cell lines PC3, DU145 and LNCaP examined. The staining was diffuse, located in the cytoplasm but not associated with any subcellular fraction. Absorption experiments indicated that this staining detected with the antibody was specific for VEGF. The staining seen was not diminished by incubation of the antibody with PDGF, TGF-α or BSA. The order of expression of VEGF as judged by intensity of staining was PC3 > DU145 > LNCaP.

### Discussion

A variety of factors have been found to influence the proliferation, migration and differentiation of endothelial cells but one of the more specific mitogens of capillary endothelium is a member of the platelet-derived growth factor (PDGF) family, vascular endothelial growth factor/vascular permeability factor. Although increased expression of this factor has been detected in several carcinomas when compared with their normal tissue counterparts (Takahashi *et al.*, 1994; O'Brien *et al.*, 1995), its presence has not previously been reported in prostate tumour specimens or prostatic cell lines.

This immunohistochemical study indicates that VEGF is present in both the benign and malignant prostate specimens, with the carcinomas having a slightly increased expression as judged by their cytoplasmic staining intensity. It would appear that a source of this particular cytokine is present within the majority of these tumours and may play a role in the neovascularisation essential for tumour growth and metastatic spread.

One of the ways in which the angiogenic activity of a tumour is assessed is by measurement of the microvessel density (MVD) in the tissue specimen by using antibodies to blood vessel markers such as factor VIII. Increased MVD has been correlated with a higher incidence of metastasis and a worse prognosis in both breast (Weidner *et al.*, 1992; Horak *et al.*, 1992) and prostatic carcinoma (Vesalainen *et al.*, 1994; Wakui *et al.*, 1992). Two studies in prostate cancer, one in invasive carcinoma (Weidner *et al.*, 1993) and the other in clinically localised disease (Hall *et al.*, 1994), concluded that MVD had prognostic potential. In non-small-cell lung carcinoma the MVD measurements also related to progression (Macchiarini *et al.*, 1992).

There is some evidence that angiogenic activity is more important at earlier stages of growth when the tumour changes from an avascular to vascular phase (Vesalainen *et al.*, 1994). Experiments on tumour transplantation into nude mice indicate that tumour take and growth are very dependent on this feature, more so than mitotic rates, and furthermore, administration of antibodies to VEGF has been shown to slow xenograft growth (Kondo *et al.*, 1993; Kim *et al.*, 1993).

It is possible that alternative angiogenic pathways may be operating in the various tumours. In bladder cancer, VEGF expression was higher in superficial tumours than invasive tumours whereas platelet-derived endothelial cell growth factor expression was higher in the invasive compared with the superficial bladder tumours (O'Brien *et al.*, 1995). In this same study VEGF expression in the superficial bladder cancer patients predicted progression.

Basic fibroblast growth factor (FGF) is also a potent mitogen of endothelial cells as well as many other cell types and is expressed in a variety of tumours (Benharroch and Birnbaum, 1990). In the transplantable human prostatic carcinoma line, Ten12, which is extremely angiogenic in nude mice, VEGF expression was not detected, but its secretion of basic FGF however was found to be 5-fold higher than that of the other prostate cell lines, PC3, DU145 and LNCaP (unpublished work). All three *in vitro* prostatic cell lines expressed VEGF as detected immunohistochemically. It is possible that the prostatic carcinomas which were negative for VEGF (15%) in this study may express alternative angiogenic compounds such as basic FGF which aid their neovascularisation. In a study of benign and malignant breast tumours higher levels of acidic and basic FGF mRNAs were found in the benign specimens although approximately 25% of the carcinomas had equally as high concentrations of basic FGF mRNA (Anandappa *et al.*, 1994). This indicates that carcinomas, within an organ type, have the ability to express and possibly use different cytokines for growth and angiogenesis.

Of interest in this current study was the observation that the neuroendocrine cells present in the prostatic tumours exhibited intense staining for VEGF. A correlation was found between the total number of chromogranin A-positive cells and the total number of VEGF-containing neuroendocrine-like cells in individual tumours. Furthermore, consecutive sections indicated that these were almost certainly the same cells (see Figure 2 a–d). These VEGF-positive neuroendocrine cells were more frequently observed in cribriform tumours which is reminiscent of the distribution of paneth-like cells described in prostate tumours (Adlakha and Bostwick, 1994).

Neuroendocrine cells were found in 55% of the carcinoma specimens in this current study which is a similar incidence to that reported by other groups (Cohen *et al.*, 1990). They express prostate-specific antigen (PSA) (Aprikian *et al.*, 1993; Cohen *et al.*, 1992) and are believed to represent a third epithelial type originating from a common stem cell. As these cells do not appear to proliferate (Bonkhoff *et al.*, 1991), it is assumed that the increased numbers seen in the tumours originate from prostatic carcinoma cells which are induced to undergo neuroendocrine differentiation. Prostatic neuroendocrine cells lack androgen receptor and it has been suggested that they could represent an androgen-insensitive cell population that can expand in some cases to populate the tumour (Krijnen *et al.*, 1993). There does, however, appear to be three types of prostatic tumours with neuroendocrine differentiation (Di Sant'Agnese, 1992) and the most common pattern, adenocarcinoma with focal neuroendocrine differentiation, is the one seen in this study. Small-cell carcinoma of the prostate is, however, prostate-specific antigen (PSA) and androgen receptor negative and, as elsewhere, is a highly expansive and aggressive tumour.

The majority of the prostatic neuroendocrine cells secrete serotonin and to lesser extents TSH, calcitonin and gastrin-releasing peptide (Abrahamsson *et al.*, 1987). Many of these peptides are thought to influence progression of the tumours via their growth-regulatory activities. More importantly the number of neuroendocrine cells in prostatic cancer correlated with poor prognosis (Cohen *et al.*, 1991; Turbat-Herrera *et al.*, 1988). The presence of a whole variety of neuropeptides and cytokines in these cells, which could act in a paracrine manner on the neighbouring cancer cell population, probably accounts for their relationship to tumour aggressiveness and progression. Increased expression of VEGF in neuroendocrine cells could also influence progression via its local angiogenic activity and, because of its action on vascular permeability, might facilitate metastatic spread. Expression of the angiogenic factors PDGF and basic FGF has been demonstrated in neuroendocrine tumours of the digestive system (Chaudhry *et al.*, 1993) and marked vascular proliferation observed in brain metastases of small-cell lung carcinomas which related to basic FGF expression (Ito *et al.*, 1993) but no evaluation of VEGF expression was made in either study.

The observation that TGF-$\alpha$ is expressed in a subset of the neuroendocrine cells would suggest that this factor may contribute to the unfavourable prognosis of the particular patient. TGF-$\alpha$, in addition to having angiogenic properties (Schreiber *et al.*, 1986), has prognostic relevance (Bebok *et al.*, 1994; Sauter *et al.*, 1995) and is also capable of stimulating prostatic cell growth in an autocrine and/or paracrine manner via the EGFR receptors which are present in many of the prostate adenocarcinomas (Harper *et al.*, 1995). Recently TGF-$\alpha$ has been reported to be expressed in all 30 neuroendocrine tumours examined, which included carcinoid tumours of the midgut, medullary thyroid carcinomas and phaeochromocytomas (Nilsson *et al.*, 1995). Large amounts of TGF-$\alpha$ were also found to be secreted into the medium by primary cultures of carcinoid tumours and phaeochromocytomas (Nilsson *et al.*, 1995). Several other growth factors, IGF-I, IGF-II, PDGF and TGF-$\beta$, have also been detected in carcinoid tumours (Beauchamp *et al.*, 1991; Chaudhry *et al.*, 1992; Nilsson *et al.*, 1992). There are reports that the cell lines PC3 and LNCaP display certain neuroendocrine characteristics expressing markers of the neural crest in addition to epithelial markers. In response to cyclic AMP analogues they were shown to differentiate terminally, with epithelial cell markers being down-regulated whereas neurone-specific enolase was elevated (Bang *et al.*, 1994). Furthermore, all three prostate cell lines have been shown to be growth inhibited by selective serotonin receptor antagonists such as pindobind (Abdul *et al.*, 1994). PC3, DU145 and LNCaP cell lines are known to express TGF-$\alpha$ (Schurmans *et al.*, 1991; Ching *et al.*, 1993; Liu *et al.*, 1993) and in the latter line the response is modulated by androgens.

It was noticed that some TGF-$\alpha$-containing neuroendocrine-like cells were negative for chromogranin A and may represent a cell subpopulation which is chromogranin B positive. In some poorly differentiated prostatic tumours chromogranin B is the major component (Schmid *et al.*, 1994).

Polymorphonuclear leucocytes were also strongly stained with VEGF antibody which appeared specific according to the absorption studies. They were noticeable in vessels adjacent to infection and/or inflammation. It is possible that the vascular permeability function of this factor may be more relevant in this situation.

In conclusion, this study indicates that the majority of prostatic tumours express VEGF which may aid their neovascularisation. The neuroendocrine cells present focally in many of the tumours appear to express VEGF in large amounts and a subpopulation of these secrete TGF-$\alpha$. In addition to their production of a variety of compounds which can act in a paracrine manner to stimulate adjacent cancer cell growth, the storage of these two angiogenic factors in such cells provides another pathway by which neuroendocrine cells could influence the progression of this disease.

**Acknowledgement**
The authors thank the Tenovus organisation for their generous financial support.

### References

ABDUL M, ANEZINIS PE, LOGOTHETIS CJ AND HOOSEIN NM. (1994). Growth inhibition of human prostate cell lines by serotonin antagonists. *Anticancer Res.*, **14**, 1215-1220.

ABRAHAMSSON PA, WADSTROM LB, ALUMETS J, FALKMER S AND GRIMELIUS L. (1987). Peptide hormone- and serotonin-immunoreactive tumour cells in carcinoma of the prostate. *Pathol. Res. Prac.*, **182**, 298-307.

ADLAKHA H AND BOSTWICK DG. (1994). Paneth cell like change in prostatic adenocarcinoma represents neuroendocrine differentiation: report of 30 cases. *Hum. Pathol.*, **25**, 135-139.

ANANDAPPA SY, WINSTANLEY JH, LEINSTER S, GREEN B, RUDLAND PS AND BARRACLOUGH R. (1994). Comparative expression of fibroblast growth factor mRNAs in benign and malignant breast disease. *Br. J. Cancer*, **69**, 772-776.

APRIKIAN AG, CORDON-CARDO C, FAIR WR AND REUTER VE. (1993). Characterization of neuroendocrine differentiation in human benign prostate and prostatic adenocarcinoma. *Cancer*, **71**, 3952-3965.

BANG YJ, PIRNIA F, FANG WG, KANG WK, SARTO O, WHITESELL L, HA MJ, TSOKOS M, SHEAHAN MD AND NGUYEN P. (1994). Terminal neuroendocrine differentiation of human prostate carcinoma cells in response to increased intracellular cyclic AMP. *Proc. Natl Acad. Sci. USA*, **91**, 5330-5534.

BEAUCHAMP RD, COFFEY RJ, LYONS RM, PERKETT EA, TOWNSEND CM AND MOSES HL. (1991). Human carcinoid cell production of paracrine growth factors that can stimulate fibroblast and endothelial cell growth. *Cancer Res.*, **51**, 5253-5260.

BEBOK Z, MARKUS B AND NEMETH P. (1994). Prognostic relevance of transforming growth factor alpha (TGF alpha) and tumor necrosis factor alpha (TNF-alpha) detected in breast cancer tissues by immunohistochemistry. *Breast Cancer Res. Treat.*, **29**, 229-235.

BENHARROCH D AND BIRNBAUM D. (1990). Biology of the fibroblast growth factor gene family. *Israel J. Med. Sci.*, **26**, 212-219.

BONKHOFF H, WERNERT N, DHOM G AND REMBERGER K. (1991). Relation of endocrine-paracrine cells to cell proliferation in normal, hyperplastic and neoplastic human prostate. *Prostate*, **19**, 91-98.

BOSARI S, LEE AK, DELELLIS RA, WILEY BD, HEATLEY GJ AND SILVERMAN ML. (1992). Microvessel quantitation and prognosis in invasive breast carcinoma. *Hum. Pathol.*, **23**, 755-761.

BROWN LF, BERSE B, JACKMAN RW, TOGNAZZI K, GUIDI AJ, DVORAK HF, SENGER DR, CONNOLLY JL AND SCHNITT SJ. (1995). Expression of vascular permeability factor (vascular endothelial growth factor) and its receptors in breast cancer. *Hum. Pathol.*, **26**, 86-91.

CHAUDHRY A, PAPANICOLAOU V, OBERG K, HELDIN CH AND FUNA K. (1992). Expression of platelet derived growth factor and its receptor in neuroendocrine tumors of the digestive system. *Cancer Res.*, **52**, 1006-1012.

CHAUDHRY A, FUNA K AND OBERG K. (1993). Expression of growth factor peptides and their receptors in neuroendocrine tumors of the digestive system. *Acta Oncol.*, **32**, 107-114.

CHING KZ, RAMSEY E, PETTIGREW N, D'CUNHA R, JASON M AND DODD JG. (1993). Expression of mRNA for epidermal growth factor, transforming growth factor alpha and their receptor in human prostate tissue and cell lines. *Mol. Cell Biochem.*, **126**, 151-158.

COHEN RJ, GLEZERSON G, HAFFEJEE Z AND AFRIKA D. (1990). Prostatic carcinoma: histological and immunohistological factors affecting prognosis. *Br. J. Urol.*, **66**, 405-410.

COHEN RJ, GLEZERSON G AND HAFFEJEE Z. (1991). Neuroendocrine cells - A new prognostic marker in prostatic cancer. *Br. J. Urol.*, **68**, 258-262.

COHEN RJ, GLEZERSON G AND HAFFEJEE Z. (1992). Prostate specific antigen and prostate specific acid phosphatase in neuroendocrine cells of prostatic cancer. *Arch. Pathol. Lab. Med.*, **116**, 65-66.

DI SANT'AGNESE PA. (1992). Neuroendocrine differentiation in human prostatic carcinoma. *Hum. Pathol.*, **23**, 287-296.

DI SANT'AGNESE PA AND DE MESEY JENSEN KL. (1987). Neuroendocrine differentiation in prostatic carcinoma. *Hum. Pathol.*, **18**, 849–856.

FOLKMAN J. (1990). What is the evidence that tumors are angiogenesis dependent? *J. Natl Cancer Inst.*, **82**, 4–6.

FOLKMAN J AND KLAGSBRUN M. (1987). Angiogenic factors. *Science*, **235**, 442–447.

GLYNNE-JONES E, HARPER ME, GODDARD L, EATON CL, MATTHEWS PN AND GRIFFITHS K. (1994). Transforming growth factor beta expression in benign and malignant prostatic tumors. *Prostate*, **25**, 210–218.

HALL MC, TRONCOSO P, POLLACK A, ZHAU HY, ZAGARS GK, CHUNG LW AND VON ESCHENBACH AC. (1994). Significance of tumor angiogenesis in clinically localised prostate carcinoma treated with external beam radiotherapy. *Urology*, **44**, 869–875.

HARPER ME, GODDARD L, GLYNNE-JONES E, WILSON DW, PRICE THOMAS M, PEELING WB AND GRIFFITHS K. (1993). An immunocytochemical analysis of TGFα expression in benign and malignant prostatic tumors. *Prostate*, **23**, 9–24.

HARPER ME, GODDARD L, GLYNNE JONES E, PEELING WB AND GRIFFITHS K. (1995). Epidermal growth factor expression by northern analysis and immunocytochemistry in benign and malignant prostatic tumours. *Eur. J. Cancer*, **31A**, 1492–1497.

HORAK ER, LEEK R, KLENK N, LEJEUNE S, SMITH K, STUART N, GREENALL M AND HARRIS AL. (1992). Angiogenesis assessed by platelet/endothelial cell adhesion molecule antibodies as an indicator of node metastasis and survival in breast cancer. *Lancet*, **340**, 1120–1124.

ITO T, KITAMURA H, NAKAMURA H, KAMEDA Y AND KANISAWA M. (1993). A comparative study of vascular proliferation in brain metastasis of lung carcinomas. *Virchows Archiv A*, **423**, 13–17.

KIM KJ, LI B, WINER J, ARMANINI M, GILLETT N, PHILLIPS HS AND FERRARA N. (1993). Inhibition of vascular endothelial growth factor-induced angiogenesis suppresses tumour growth in vivo. *Nature*, **362**, 841–844.

KONDO S, ASANO M AND SUZUKI H. (1993). Significance of vascular endothelial growth factor/permeability factor for solid tumor growth, and its inhibition by the antibody. *Biochem. Biophys. Res. Commun.*, **194**, 1234–1241.

KRIJNEN JLM, JANSSEN PJA, RUIZEVELD DE WINTER JA, VAN KRIMPEN H, SCHOEDER FH AND VAN DER KWAST TH. (1993). Do neuroendocrine cells in human prostate cancer express androgen receptor? *Histochemistry*, **100**, 393–398.

LIU XH, WILEY HS AND MEIKLE AW. (1993). Androgens regulate proliferation of human prostate cancer cells in culture by increasing transforming growth factor-alpha (TGF-alpha) and epidermal growth factor (EGF)/TGF-alpha receptor. *J. Clin. Endocrinol. Metab.*, **77**, 1472–1478.

MACCHIARINI P, FONTANINI G, HARDIN MJ, SQUARTINI F AND ANGELETTI CA. (1992). Relation of neovascularization to metastasis of non-small-cell lung cancer. *Lancet*, **340**, 145–146.

MATTERN J, KOOMAGI R AND VOLM M. (1996). Association of vascular endothelial growth factor expression with intratumoral microvascular density and tumour cell proliferation in human epidermoid lung carcinoma. *Br. J. Cancer*, **73**, 931–934.

MOSTOFI FK, SESTERHOLM I AND SOBIN LH. (1980). Histological typing of prostatic tumours. *International Histological Classification of Tumours*, **No. 22.** World Health Organization: Geneva.

NEUFELD G, TESSLER S, GITAY-GOREN H, COHEN T AND LEVI BZ. (1994). Vascular endothelial growth factor and its receptors. *Prog. Growth Factor Res.*, **5**, 89–97.

NILSSON O, WANGBERG B, THEODORSSON E, SKOTTNER A AND AHLMAN H. (1992). Presence of IGF-I in human midgut carcinoid tumours – an autocrine regulator of carcinoid tumour growth? *Int. J. Cancer*, **51**, 195–203.

NILSSON O, WANGBERG B, KOLBY L, SCHULTZ GS AND AHLMAN H. (1995). Expression of transforming growth factor alpha and its receptor in human neuroendocrine tumours. *Int. J. Cancer*, **60**, 645–651.

O'BRIEN T, CRANSTON D, FUGGLE S, BICKNELL R AND HARRIS AL. (1995). Different angiogenic pathways characterise superficial and invasive bladder cancer. *Cancer Res.*, **55**, 510–513.

ONO M, OKAMURA K, NAKAYAMA Y, TOMITA M, SATO Y, KOMATSU Y AND KUWANO M. (1992). Induction of human microvascular endothelial tubular morphogenesis by human keratinocytes: involvement of transforming growth factor-alpha. *Biochem. Biophys. Res. Commun.*, **189**, 601–609.

PLATE KH, BREIER G AND RISAU W. (1994). Molecular mechanisms of developmental and tumor angiogenesis. *Brain Pathol.*, **4**, 207–218.

REINARTZ JJ, GEORGE E, LINDGREN BR AND NIEHANS GA. (1994). Expression of p53, transforming growth factor alpha, epidermal growth factor receptor and c-*erb*B-2 in endometrial carcinoma and correlation with survival and known predictors of survival. *Hum. Pathol.*, **25**, 1075–1083.

SAUTER ER, COIA LR, EISENBERG BL AND HANKS GE. (1995). Transforming growth factor alpha expression as a potential survival prognosticator in patients with oesophageal adenocarcinoma receiving high dose radiation and chemotherapy. *Int. J. Rad. Oncol. Biol. Phys.*, **31**, 567–569.

SCHMID KW, HELPAP B, TOTSCH M, KIRCHMAIR R, DOCKHORN-DWORNICZAK B, BOCKER W AND FISCHER-COLBRIE R. (1994). Immunohistochemical localization of chromogranins A and B and secretogranin II in normal, hyperplastic and neoplastic prostate. *Histopathology*, **24**, 233–239.

SCHREIBER AB, WINKLER ME AND DERYNCK R. (1986). Transforming growth factor α: a more potent angiogenic mediator than epidermal growth factor. *Science*, **232**, 1250–1253.

SCHUURMANS AL, BOLT J, VELDSCHOLTE J AND MULDER E. (1991). Regulation of the growth of LNCaP tumor cells by growth factors and steroid hormones. *J. Steroid Biochem. Mol. Biol.*, **40**, 193–197.

TAKAHASHI A, SASAKI H, KIM SJ, TOBISU K, KAKIZOE T, TSUKAMOTO T, KUMAMOTO Y, SUGIMURA T AND TERADA M. (1994). Markedly increased amounts of messenger RNAs for vascular endothelial growth factor and placental growth factor in renal cell carcinoma associated with angiogenesis. *Cancer Res.*, **54**, 4233–4237.

TOI M, HOSHINA S, TAKAYANAGI T AND TOMINAGA T. (1994). Association of vascular endothelial growth factor expression with tumor angiogenesis and with early relapse in primary breast cancer. *Jpn. J. Cancer Res.*, **85**, 1045–1049.

TOI M, KONDO S, SUZUKI H, YAMAMOTO Y, INADA K, IMAMAWA T, TANIGUCHI T AND TOMINAGA T. (1996). Quantitative analysis of vascular endothelial growth factor in primary breast cancer. *Cancer*, **77**, 1101–1106.

TURBAT-HERRERA EA, HERRERA GA, GORE I, LOTT RL, GRIZZLE WE AND BONNIN JM. (1988). Neuroendocrine differentiation in prostatic carcinoma. A retrospective autopsy study. *Arch. Pathol. Lab. Med.*, **112**, 1100–1105.

VESALAINEN S, LIPPONEN P, TALJA M, ALHAVA E AND SYRJANEN K. (1994). Tumor vascularity and basement membrane structure as prognostic factors in T12MO prostatic adenocarcinoma. *Anticancer Res.*, **14**, 709–714.

VUKANOVIC J, HARTLEY-ASP B AND ISAACS JT. (1995). Inhibition of tumor angiogenesis and the therapeutic ability of linomide against rat prostatic cancer. *Prostate*, **26**, 235–246.

WAKUI S, FURUSATO M, ITOH T, SASAKI H, AKIYAMA A, KINOSHITA I, ASANO K, TOKUDA T, AIZAWA S AND USHIGOME S. (1992). Tumour angiogenesis in prostatic carcinoma with and without bone metastasis: a morphological study. *J. Pathol.*, **168**, 257–262.

WEIDNER N, FOLKMAN J, POZZA F, BEVILACQUA P, ALLRED EN, MOORE DH, MELI S AND GASPARINI G. (1992). Tumor angiogenesis. A new significant and independent prognostic indicator in early stage breast carcinoma. *J. Natl Cancer Inst.*, **84**, 1875–1887.

WEIDNER N, CARROLL PR, FLAX J, BLUMENFELD W AND FOLKMAN J. (1993). Tumor angiogenesis correlates with metastasis in invasive prostate carcinoma. *Am. J. Pathol.*, **143**, 401–409.

WEINDEL K, MORINGLANE JR, MARME D AND WEICH HA. (1994). Detection and quantification of vascular endothelial growth factor/vascular permeability factor in brain tumor tissue and cyst fluid: key to angiogenesis. *Neurosurgery*, **35**, 439–449.

YAMAMOTO T, TERADA N, NISHIZAWA Y AND PETROW V. (1994) Angiostatic activities of medroxyprogesterone acetate and its analogues. *Int. J. Cancer*, **56**, 393–399.

YAMAZAKI K, ABE S, TAKEKAWA H, SUKOH N, WATANABE N, OGURA S, NAKAJIMA I, ISOBE H, INOUE K AND KAWAKAMI Y. (1994). Tumor angiogenesis in human lung adenocarcinoma. *Cancer*, **74**, 2245–2250.

ZAGZAG D. (1995). Angiogenic growth factors in neural embryogenesis and neoplasia. *Am. J. Pathol.*, **146**, 293–309.